# PROVIDING AND RECEIVING FAMILY CARE ACROSS THE LIFE COURSE: INSIGHTS FROM THE OLDEST OLD

**DOI:** 10.1093/geroni/igz038.2279

**Published:** 2019-11-08

**Authors:** Taylor Patskanick, Julie Miller, Chaiwoo Lee, Lisa D’Ambrosio

**Affiliations:** 1 Massachusetts Institute of Technology, Cambridge, Massachusetts, United States; 2 MIT AgeLab, Cambridge, Massachusetts, United States

## Abstract

Unprecedented longevity comes with an increased need for providing and receiving care. A 2015 report estimated 39.8 million adults in the United States provided unpaid care to an adult in 2014 (NAC & AARP). Previous research has focused disproportionately on experiences of providing care to older adults, but little has explored experiences of providing care and receiving care among the oldest old. Adults aged 85 and older are likely to have provided care to an adult family member at some point in their lives, but now may be receiving care themselves. The presentation will report on findings from a mixed methods study investigating the experiences of providing and receiving care across the life course among a sample of the “oldest old.” Data draw from focus groups and a survey with the MIT AgeLab Lifestyle Leaders, a bimonthly panel study of adults ages 85 and older. Findings suggest the Lifestyle Leaders had extensive experience providing care, particularly in older age. They most often cared for family members with long-term physical or cognitive conditions. Opinions on learning new technologies to help with caregiving and robot caregivers were mixed. The majority of the Lifestyle Leaders received regular help with at least one care task regardless of household composition or living situation. Many reported help had improved their health, but they felt like a burden to their caregivers. Even in later life, the Lifestyle Leaders had few ideas about who might take care of them if they needed care in the future.

